# Réponse radiologique complète d'un hémangiopéricytome méningé après radiothérapie adjuvante à une exérèse incomplète

**DOI:** 10.11604/pamj.2014.17.150.3795

**Published:** 2014-03-03

**Authors:** Amine Bazine, Etienne Ogandaga, Serpos Dossou, Abdellah Aissa, Jamal Oumellal, Issam Lalya, Amar Saidi, Tayeb Kebdani, Noureddine Benjaafar

**Affiliations:** 1 Service de Radiothérapie-Oncologie de l'Institut National d'Oncologie, Université Mohamed V Souissi, Rabat, Maroc; 2 Service de Neurochirurgie de l'Hôpital des Spécialités, Université Mohamed V Souissi, Rabat, Maroc; 3 Centre d'Anatomie Pathologique les Nations Unies, Rabat, Maroc

**Keywords:** Hémangiopéricytome méningé, radiothérapie adjuvant, exérèse, tumeur vasculaire, hemangiopericytoma, adjuvant radiotherapy, resection, vascular tumor

## Abstract

L'hémangiopéricytome méningé (HM) est une tumeur vasculaire rare qui représente moins de 1% des tumeurs intracrâniennes. Son traitement reste à ce jour mal codifié et n'est basé que sur des données rétrospectives. La place de la radiothérapie adjuvante après chirurgie d'exérèse n'est pas bien établie. Ce présent travail décrit un cas d'HM chez une patiente de 42 ans, traité par chirurgie incomplète et radiothérapie adjuvante, avec réponse radiologique complète notée 1 an après la fin du traitement.

## Introduction

L'hémangiopéricytome est une tumeur mésenchymateuse hyper-vasculaire ubiquitaire, qui prend naissance à partir des péricytes de Zimmerman entourant les vaisseaux sanguins et les capillaires. Sa localisation méningée est rare, et se caractérise par son potentiel malin, son taux de récidive élevé et par la fréquence non négligeable de métastases à distance. En l'absence de larges essais prospectifs, la décision thérapeutique est basée généralement sur des données rétrospectives. Le rôle de la radiothérapie postopératoire n'est pas clairement établi. Elle est souvent recommandée après une chirurgie incomplète. Nous rapportons à travers cette observation un cas d'hémangiopéricytome méningé (HM) traité par chirurgie incomplète et radiothérapie adjuvante, avec une réponse radiologique complète notée 1 an après. Nous discuterons la place de la radiothérapie postopératoire à travers une revue de littérature.

## Patient et observation

**Figure 1:**
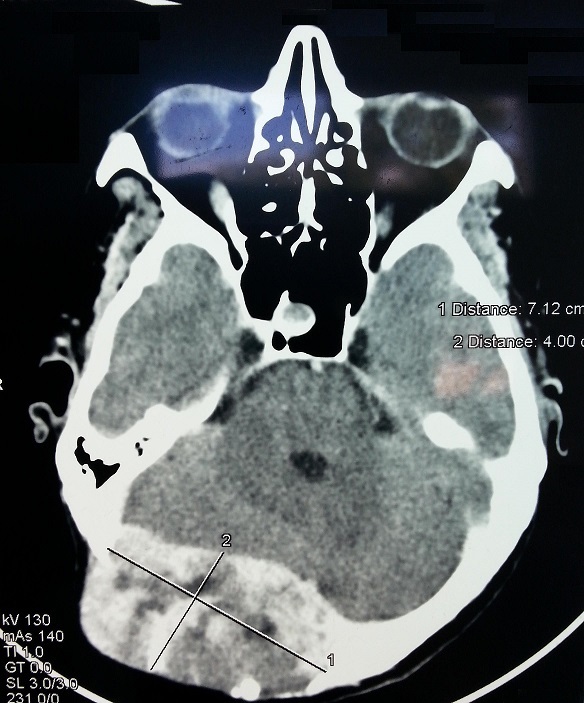
Coupe scannographique axiale montrant un processus lésionnel centré sur l’écaille occipitale, latéralisé à droite, accompagné d'une infiltration méningée

Il s'agit d'une patiente de 42 ans, sans antécédents pathologiques notables, qui s'est présentée en consultation de neurochirurgie pour une tuméfaction occipitale droite évoluant depuis une année, aves des céphalées et des troubles intermittents de la marche. La tomodensitométrie (TDM) cérébrale a objectivé un processus lésionnel centré sur l’écaille occipitale, latéralisé à droite, mesurant 71 mm sur 40 mm, refoulant le sinus veineux latéral droit, accompagné d'une infiltration méningée et entrainant un discret effet de masse sur l'hémisphère cérébelleux droit (Figure 1).

**Figure 2:**
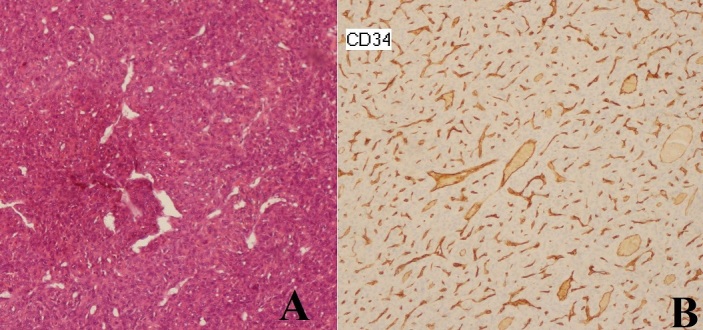
A. Coupe histologique montrant une prolifération tumorale faite de cellules aux noyaux arrondis avec une vascularisation branchée de type hémangiopéricytaire (hématoxyline éosine ×200). B. Les cellules expriment de façon diffuse et intense l'antigène CD34 (G ×200)

Une exérèse chirurgicale en fragments de l’écaille occipitale a été réalisée. L'examen histologique montre un processus tumoral de nature mésenchymateuse et de forte densité cellulaire. Cette prolifération tumorale est faite de cellules aux noyaux arrondis avec un cytoplasme peu abondant. Les atypies cytonucléaires sont modérées et l'activité mitotique est faible. La vascularisation est branchée de type hémangiopéricytaire. Les cellules tumorales expriment le CD34. Le diagnostic retenu est celui d'un hémangiopéricytome de grade III de l'organisation mondiale de la santé (OMS) (Figure 2).

**Figure 3:**
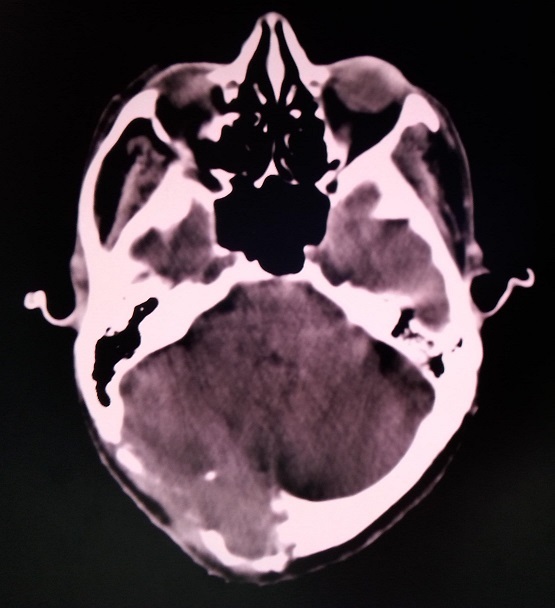
Coupe scannographique axiale montrant la persistance du processus après la chirurgie d'exérèse

Une TDM cérébrale réalisée en postopératoire a montré la persistance du processus lésionnel centré sur l'os occipital et infiltrant les méninges, mesurant cette fois ci 49 sur 30 mm (Figure 3). Un bilan d'extension à distance fait d'une TDM thoraco-abdominale et d'une scintigraphie osseuse a permis d’écarter des localisations secondaires.

**Figure 4:**
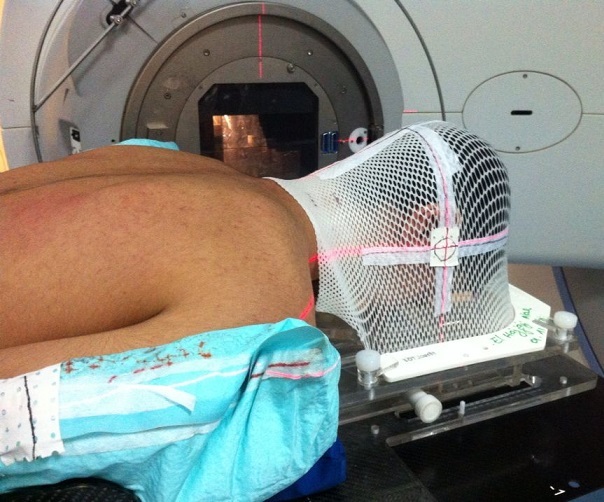
Photographie montrant la position de la patiente au cours de son traitement par radiothérapie

En réunion de concertation pluridisciplinaire, il a été décidé de réaliser une radiothérapie sur la masse résiduelle. Pour cela, la patiente était placée en décubitus ventral à l'aide d'un support pour irradiation cérébro-spinale. La tête était contenue par un masque thermoformé (Figure 4). L'acquisition des données anatomiques était réalisée par le moyen d'un scanner dosimétrique. Les images scannographiques étaient transférées vers un système de planification de traitement pour radiothérapie.

**Figure 5:**
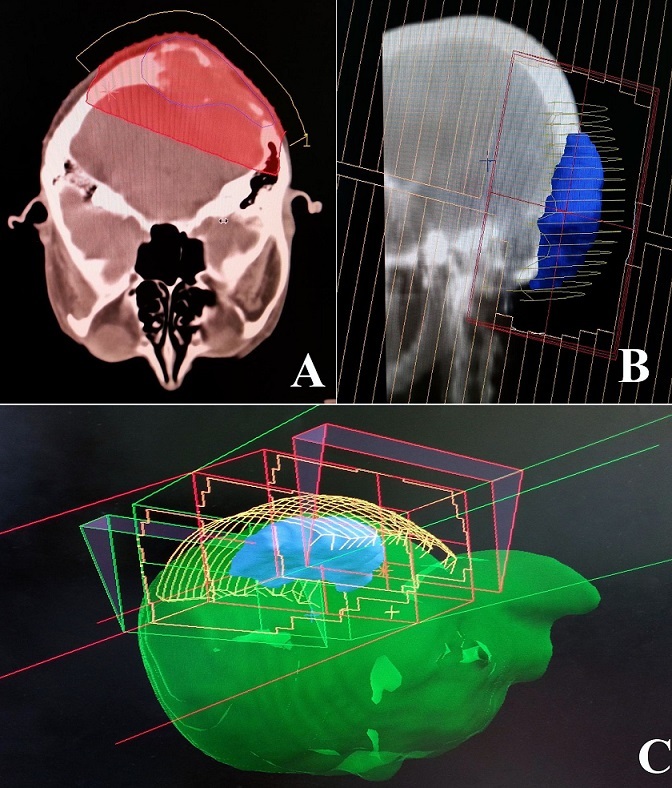
Images issues du logiciel de planification de traitement pour radiothérapie. A. Coupe scannographique axiale montrant le volume cible (Contour bleu) couvert par l'isodose 95% (Aire rouge). B. Radiographie reconstruite montrant un des deux champs de traitement. C. Modèle 3D généré montrant la balistique du traitement

La tumeur macroscopique était contourée sur chaque coupe. Une reconstruction en 3D était alors générée et un plan de traitement a été proposé. La dose totale prescrite était de 60 Gray (Gy) avec un fractionnement de 2Gy/fraction, une fraction par jour, 5jour/7jours. Une balistique par deux champs opposés a été utilisée (Figure 5). Le traitement s'est étalé sur 44 jours. Une alopécie modérée par plaques était notée en cours d'irradiation.

**Figure 6:**
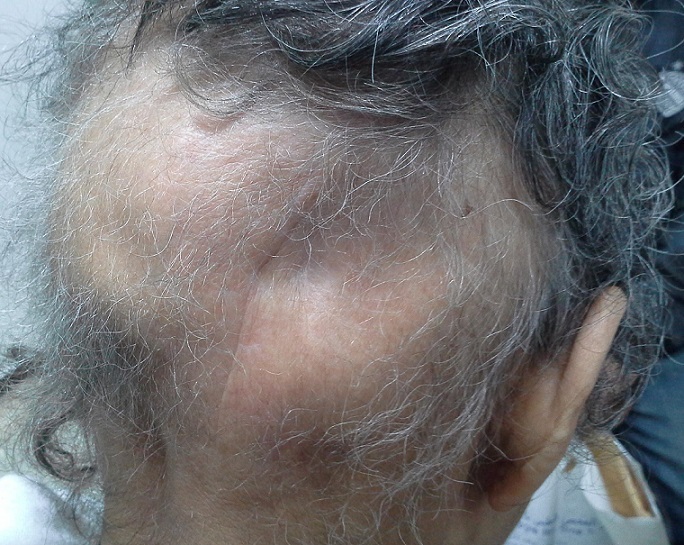
Photographie montrant la persistance d'une dépression de la voûte crânienne avec une alopécie définitive en regard de la zone irradiée

**Figure 7:**
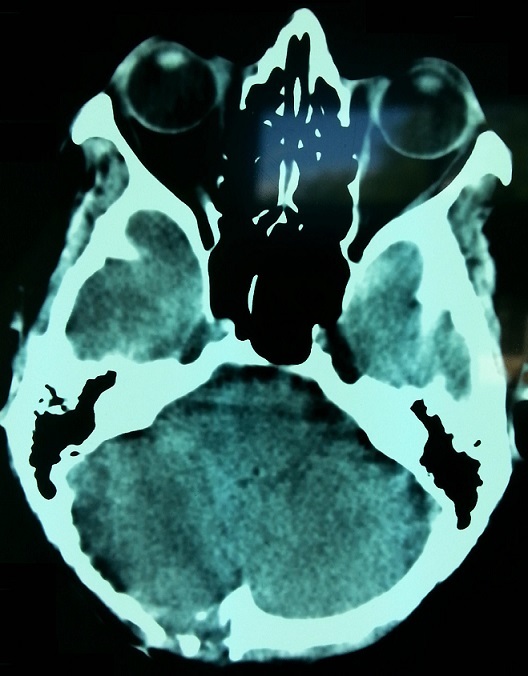
Coupe scannographique axiale montrant la disparition complète du processus tumoral

La patiente est régulièrement suivie en consultation de contrôle. Elle garde une discrète dépression de la voute crânienne avec une alopécie définitive au niveau de la zone irradiée (Figure 6). La TDM cérébrale de contrôle réalisée 1 an après la fin d'irradiation a objectivé une disparition complète du processus tumoral avec solution de continuité de l'os occipital (Figure 7).

## Discussion

Décrit pour la première fois en 1928 par Bailey [1], l'HM est une tumeur rare qui représente moins de 1% des tumeurs intracrâniennes et2 à 2,4% de l'ensemble des tumeurs méningées. Il survient principalement chez l'adulte jeune entre 38 et 47 ans, avec une discrète prépondérance masculine [2].

Initialement l'HM était considéré comme une variété appartenant aux méningiomes et portait l'appellation de méningiome angioblastique. En 1993, l'OMS a reconnu l'HM comme une entité distincte [3] et a établi en 2007 des critères clairs de classification de cette tumeur [4]. La résection macroscopique complète est le traitement de choix permettant d'obtenir une meilleure survie sans récidive, mais elle peut être impossible en raison de l'extension aux sinus duraux, de l'envahissement cérébral, de l'hyper-vascularisation ou de l'inaccessibilité anatomique de la tumeur [5, 6].

L'HM est considéré comme une tumeur relativement radiosensible [7]. Il est spéculé que la radiothérapie adjuvante après chirurgie soit supérieure à la chirurgie seule [5]. Cependant, une méta-analyse de 194 patients remet en question l'efficacité de la radiothérapie en termes de survie [6]. A noter que cette méta-analyse a inclus des données datant de plus de 50 ans et ses résultats doivent être interprétés avec prudence. Une analyse récente des cas d'HM de la base de données SEER (The Surveillance, Epidemiology, and End Results), de 1990 à 2008, a conclu que la radiothérapie améliore la survie sans récidive après chirurgie incomplète [8]. Après une résection macroscopique complète, la radiothérapie ne semble pas améliorer de façon significative la survie [6, 8]. La dose prescrite est très controversée [6, 7, 9]. En général, elle devra être comprise entre 50 et 60 Gray (Gy) [8]. Une technique conformationnelle avec des faisceaux réduits est souhaitable pour préserver les tissus sains [10].

L'HM est caractérisé par un comportement clinique agressif avec des taux de récidive rapportés allant jusqu’à 90% dans les sept mois après le traitement initial [2]. En raison de la rareté relative des HM, il est difficile de déduire les facteurs pronostiques qui influencent la survie. La réponse radiologique complète, comme c'est le cas pour notre patiente, n'a jamais été décrite comme un facteur améliorant la survie sans récidive [6].

## Conclusion

La rareté relative et la récente reclassification de l'HM expliquent l'absence de standards thérapeutiques de cette tumeur. La radiothérapie adjuvante est indiquée après une résection incomplète. Une technique conformationnelle avec des faisceaux réduits est privilégiée avec des doses variant entre 50 et 60 Gy. La réponse radiologique n'est pas rapportée dans la littérature comme un facteur influençant la survie.

## References

[1] Bailey P, Cushing H, Eisenhardt L (1928). Angioblastic meningiomas. Arch Pathol..

[2] Schiariti M, Goetz P, El-Maghraby H (2011). Hemangiopericytoma: long-term outcome revisited. J Neurosurg..

[3] Kleihues P, Burger PC, Scheithauer BW Histological typing of tumours of the Central Nervous System.

[4] Louis DN, Ohgaki H, Wiestler OD (2007). The 2007 WHO classification of tumours of the central nervous system. Acta Neuropathol..

[5] Rutkowski MJ, Jian BJ, Bloch O (2012). Intracranial hemangiopericytoma: Clinical experience and treatment considerations in a modern series of 40 adult patients. Cancer..

[6] Rutkowski MJ, Sughrue ME, Kane AJ (2010). Predictors of mortality following treatment of intracranial hemangiopericytoma. J Neurosurg..

[7] Dufour H, Métellus P, Fuentes S (2001). Meningeal hemangiopericytoma: a retrospective study of 21 patients with special review of postoperative external radiotherapy. Neurosurgery..

[8] Stessin AM, Sison C, Nieto J (2013). The Role of Postoperative Radiation Therapy in the Treatment of Meningeal Hemangiopericytoma: Experience From the SEER Database. Int J Radiation Oncol Biol Phys..

[9] Guthrie BL, Ebersold MJ, Scheithauer BW (1989). Meningeal hemangiopericytoma: histopathological features, treatment, and long-term follow-up of 44 cases. Neurosurgery..

[10] Combs SE, Thilmann C, Debus J (2005). Precision radiotherapy for hemangiopericytomas of the central nervous system. Cancer..

